# A TSPO ligand attenuates brain injury after intracerebral hemorrhage

**DOI:** 10.1096/fj.201601377RR

**Published:** 2017-04-17

**Authors:** Minshu Li, Honglei Ren, Kevin N. Sheth, Fu-Dong Shi, Qiang Liu

**Affiliations:** * Department of Neurology, Tianjin Neurological Institute, Tianjin Medical University General Hospital, Tianjin, China;; † Department of Neurology, Barrow Neurological Institute, St. Joseph’s Hospital and Medical Center, Phoenix, Arizona, USA;; ‡ Department of Neurology, Yale University School of Medicine, New Haven, Connecticut, USA

**Keywords:** immune modulation, etifoxine, hemorrhagic stroke, inflammation

## Abstract

Intracerebral hemorrhage (ICH) is a devastating disease without effective treatment. After ICH, the immediate infiltration of leukocytes and activation of microglia are accompanied by a rapid up-regulation of the 18-kDa translocator protein (TSPO). TSPO ligands have shown anti-inflammatory and neuroprotective properties in models of CNS injury. In this study, we determined the impact of a TSPO ligand, etifoxine, on brain injury and inflammation in 2 mouse models of ICH. TSPO was up-regulated in Iba1^+^ cells from brains of patients with ICH and in CD11b^+^CD45^int^ cells from mice subjected to collagenase-induced ICH. Etifoxine significantly reduced neurodeficits and perihematomal brain edema after ICH induction by injection of either autologous blood or collagenase. In collagenase-induced ICH mice, the protection of etifoxine was associated with reduced leukocyte infiltration into the brain and microglial production of IL-6 and TNF-α. Etifoxine improved blood–brain barrier integrity and diminished cell death. Notably, the protective effect of etifoxine was abolished in mice depleted of microglia by using a colony-stimulating factor 1 receptor inhibitor. These results indicate that the TSPO ligand etifoxine attenuates brain injury and inflammation after ICH. TSPO may be a viable therapeutic target that requires further investigations in ICH.—Li, M., Ren, H., Sheth, K. N., Shi, F.-D., Liu, Q. A TSPO ligand attenuates brain injury after intracerebral hemorrhage.

Intracerebral hemorrhage (ICH) is a devastating condition that afflicts 10–15% of all stroke victims, but no effective treatment exists (1–5). Emerging evidence suggests that inflammation plays a key role in the progression of ICH-induced brain injury (4, 6, 7). Upon the onset of ICH, blood components, including leukocytes, enter the brain and activate resident immune cells, such as microglia. Subsequently, the infiltrating leukocytes and activated microglia then augment the local production of proinflammatory cytokines. Together with cell death products, these factors amplify blood–brain barrier (BBB) disruption and destroy surrounding tissues, contributing to the development of perihematomal edema and the aggravated mass effect (4, 6, 7). Therefore, brain inflammation has emerged as a major modifiable determinant as a target for the development of new ICH treatment (7).

The 18-kDa translocator protein (TSPO) is a 5-transmembrane unit localized on the outer mitochondrial membrane with the major function of transporting cholesterol across that membrane for neurosteroid synthesis (8). As leukocytes infiltrate and microglia become activated after ICH, TSPO is rapidly up-regulated in microglia and other brain-resident immune cells (9). Substantial evidence has verified the augmentation of TSPO in several neuroinflammatory conditions, including ischemic stroke (10, 11) and multiple sclerosis (12, 13). The prevalence and up-regulation of TSPO in the neuroinflammatory environment suggest that targeting TSPO may be a viable approach to limiting neuroinflammation and brain injury after ICH. In a recent study of experimental ICH, the up-regulation of TSPO was documented in brain microglia (9), but still unclear is what immune cell types may express TSPO after ICH.

Etifoxine is a clinically available, high-affinity TSPO ligand (8). Previous studies have shown beneficial effects of etifoxine and other TSPO ligands in down-regulating microglial activation and promoting neural survival after several types of CNS injury (12, 14–16). However, no studies have been performed to determine the potential impact of etifoxine on brain injury and inflammation in the context of ICH. In this study, in 2 mouse models of ICH, etifoxine treatment attenuated neurodeficits, lesion size, perihematomal edema, cell death, and neuroinflammation.

## MATERIALS AND METHODS

### Human brain tissue

Human brain tissues were obtained at Tianjin Medical University General Hospital. The protocols were approved by the institutional review boards in Tianjin Medical University General Hospital. Among the 9 patients studied, the perihematomal tissues were from 5 patients who underwent surgical evacuation of hematomas within 24 h of ICH onset. The specimens were collected with informed consent. The patients with ICH were between 40 and 60 yr of age with a computed tomography-confirmed hematoma located in the basal ganglia (30–70 ml). The other 4 samples were obtained from individuals who died of nonneurologic diseases, and those tissues were collected within 4 h after death for use as controls. The 4 control subjects had no history of neurologic or neuropsychiatric diseases, which was confirmed by histopathological examination.

### Animals

All animal experiments were approved by the Committee on the Ethics of Animal Experiments of Tianjin Neurologic Institute and Barrow Neurologic Institute. All experiments were performed in accordance with the *Guide for the Care and Use of Laboratory Animals in China* and *Guide for the Care and Use of Laboratory Animals* [U.S. National Institutes of Health (NIH), Bethesda, MD, USA]. Male C57BL/6 mice (Charles River Laboratories, Wilmington, MA, USA), 8 to 10 wk old, were used. The mice were randomly assigned into each experimental group. All mice were housed in pathogen-free conditions of the vivarium facilities. All surgeries were performed with animals under anesthesia. Reporting of this study complies with the Animal Research: Reporting *In Vivo* Experiments (ARRIVE) guidelines (*https://www.nc3rs.org.uk/arrive-guideline*s).

### Induction of ICH in mice

ICH was induced as we have described (17–20). Mice were anesthetized with a cocktail of ketamine (100 mg/kg) and xylazine (10 mg/kg) by intraperitoneal injection. Thereafter, they were fixed on a stereotactic frame. A hole was drilled on the right side of the skull (2.3 mm lateral to midline, 0.5 mm anterior to bregma). For the ICH model induced by collagenase injection, 0.0375 U bacterial collagenase (type IV-S; Sigma-Aldrich, St. Louis, MO, USA) in 0.5 μl saline was infused at a rate of 1 μl/min at the caudate nucleus (3.7-mm depth below the surface of the skull) through an infusion pump (KD Scientific, Holliston, MA, USA). For the ICH model induced by injection of autologous blood, a double-injection method was used. Whole blood (30 μl) withdrawn from the angular vein was transferred into a 50 μl syringe with a 26-gauge needle (Hamilton Company, Reno, NV, USA). The needle was inserted into the right caudate nucleus (2.3 mm lateral to the midline; 0.5 mm anterior to the bregma; 3 mm below the surface of the skull). During the infusion process, the first 5 μl of blood was injected at the rate of 1 μl/min through the infusion pump beneath the hole, to generate a clot; the needle was then moved to a depth of 3.7 mm from the 3-mm position. After a 5-min pause, the remaining 25 μl blood was infused at the same rate of 1 μl/min. The needle was held in position for an additional 20 min and then gently withdrawn. Sham-treated controls were injected with an equal volume of saline. The burr hole was sealed with bone wax (Johnson & Johnson, New Brunswick, NJ, USA). During surgery, body temperatures were maintained at 37 ± 0.5°C. Afterward, animals were placed in cages and given free access to food and water.

### Drug administration

Etifoxine was dissolved in saline supplement with 1% Tween-80 as previously described (12). Etifoxine or the saline vehicle was administrated at 50 mg/kg in 200 μl by intraperitoneal injection immediately after ICH and was given daily until the experiment ended.

### Neurologic assessment

Neurologic deficits were assessed at d 1 and 3 after ICH by at least 2 investigators blinded to the treatments of all ICH mice in each group. The modified Neurologic Severity Score (mNSS) and corner-turning test were performed as described (21, 22). The mNSS rates neurologic functioning and includes a composite of motor, sensory, reflex, and balance tests. The corner-turning test was used to assess sensorimotor and postural asymmetries. The mouse being tested was allowed to enter a corner with an angle of 30° and was required to turn either to the left or the right to exit the corner. The clinical scoring and corner test procedures were performed as published elsewhere (23, 24).

### Neuroimaging

Three days after ICH, a 7-T small-animal MRI scan was performed to evaluate lesion volume and hematoma volume (17). T2-weighted images (repetition time, 4500 ms; echo time, 65.5 ms; field of view, 28 × 28; image matrix, 256 × 256; slice thickness, 0.5 mm) and susceptibility weighted images (SWIs; repetition time, 30 ms; echo time, 10 ms; field of view, 32 × 32; image matrix, 256 × 256; slice thickness, 0.3 mm) were acquired to assess lesion volume and hematoma volume, respectively. The lesion and hematoma areas were manually outlined on each slice, then the areas in all slices were summed and multiplied by section thickness using Medical Image Processing, Analysis, and Visualization software (MIPAV; NIH). The lesion and hematoma volumes were measured by 2 investigators blinded to the tissues source.

The T1 postcontrast images were used to assess BBB integrity (25). After administration of the contrast agent (gadopentetate dimeglumine; Gd-DTPA), (Magnevist, Schering AG, Berlin, Germany) with a dosage of 0.2 mmol/kg body weight, T1 postcontrast images were acquired with the following parameters: repetition time, 322 ms; echo time, 10.5 ms; field of view, 28 mm; image matrix, 256 × 256; slice thickness, 0.5 mm). MRI data were analyzed with MED×3.4.3 software (Medical Numerics, Germantown, MA, USA) on a Linux workstation.

### Brain water content

Brain water content was measured 3 d after ICH (26). In brief, after anesthesia and decapitation, brains of mice were obtained and divided into 3 parts: ipsilateral hemisphere, contralateral hemisphere, and cerebellum. The tissues were then weighed to obtain the wet weight, followed by drying for 24 h at 100°C to obtain the dry weight. The calculation formula of brain water contents was: (wet weight – dry weight)/wet weight × 100%.

### Flow cytometry

To analyze immune cell infiltration and microglia cytokine expression in brains, we isolated cellular components from brain tissue to perform flow cytometry analysis. Three days after ICH, brains were harvested and homogenized with 40 μm nylon cell strainers (BD Biosciences, Franklin Lakes, NJ, USA) in PBS. Cell suspensions were centrifuged at 2000 rpm for 5 min, and cell pellets were collected. Thereafter, 5 ml of 70% Percoll solution (GE Healthcare Bio Science AB, Uppsala, Sweden) was used to resuspend the cell pellet, and 30% Percoll solution was overlaid on it. The gradient was centrifuged at 2000 rpm for 30 min at room temperature. Single cells in the interface between 30 and 70% Percoll were collected for antibody staining. Cells were diluted to 1×10^6^ cells in 100 μl PBS solution with 1% bovine serum albumin and stained for antibodies and isotype control. All antibodies were purchased from BioLegend (San Diego, CA, USA), unless otherwise indicated, and the staining protocol followed the manual’s instructions. The following antibodies were used: CD45 (30-F11), CD11b (M1/70), CD3 (145-2C11), CD4 (GK1.4), CD8 (53-6.72), anti-NK1.1 (PK136), anti-CD19 (1D3), F4/80 (6F12), Ly6G (1A8). For intracellular staining, cells were fixed and permeabilized with a commercial solution (BioLegend) and then stained for the antibodies: IL-6 (MP5-20F3), IL-10 (JES5-16E3), TNF-α (MP6-XT22), TGF-β (TW7-20B9), and peripheral-type benzodiazepine receptor (PBR; Abcam, Cambridge, MA, USA) and Alexa Fluor 488-labeled donkey anti-rabbit IgG secondary antibody (Thermo Fisher Scientific, Carlsbad, CA, USA) for TSPO. Fluorescence minus one controls were stained, respectively.

To analyze cell death after ICH, we harvested single-cell suspensions as previously described and removed the myelin with 30% Percoll solution. The pellets were collected and stained for annexin V, as detailed in the manual’s protocol. Flow cytometry data were obtained on FACS Aria III (BD Biosciences) and analyzed by Flow Jo, v.7.6.1 (Informer Technologies, Walnut Creek, CA, USA).

### Histology

Brain tissues of patients with ICH and ICH mice were fixed in 4% paraformaldehyde and embedded in paraffin. Brain sections (5 µm thick) were made and rehydrated in a series of ethanol dilutions. Brain sections were permeabilized and incubated in blocking solution (5% goat or donkey serum in PBS solution), followed by incubation with primary antibodies: goat anti-Iba1 (Wako, Richmond, VA, USA) and rabbit anti-PBR (Abcam, Cambridge, MA, USA) at 4°C overnight. After triple washings with PBS, the slices were incubated with secondary antibodies: donkey anti-rabbit 594 (Thermo Fisher Scientific) and donkey anti-goat 488 (Thermo Fisher Scientific). After triple washings with PBS, the brain sections were stained with DAPI (Abcam). For TUNEL staining, the Terminal Deoxynucleotidyl Transferase Biotin-dUTP Nick End Labeling kit (Roche, Indianapolis, IN, USA) was used according to the manufacturer’s protocol. Images were captured with a microscope (Model BX-61; Olympus, Center Valley, PA, USA), and data were analyzed with Image J (NIH). For cell counting, positively stained cells were counted in 3 comparable, randomly selected microscopic fields. The numbers of cells from 9 locations per mouse (3 fields per section × 3 sections per mouse) were averaged and expressed as positive cells per field.

### Western blot analysis

Western blot analysis was performed to detect the expression of tight junction proteins as has been described (17). In brief, 3 d after ICH, ipsilateral hemispheres of brains were harvested. Proteins were extracted, electrophoresed, and transferred onto a PVDF membrane (Merck KGaA, Darmstadt, Germany). After being blocked, the PVDF membranes were incubated with primary antibodies: anti-zonula occluden-1 (ZO-1, 1:1000; Thermo Fisher Scientific); anti-claudin-5 (1:1000; Thermo Fisher Scientific) antibodies or anti-β-actin (1:1000; Cell Signaling Technology, Danvers, MA, USA) at 4°C overnight, followed by incubation with horseradish peroxidase–labeled anti-rabbit secondary antibody (1:4000; Zymed, Carlsbad, CA, USA) (27). The intensity of blots was detected with a Bio-Rad 721BR08844 Gel Doc Imager (Bio-Rad, Hercules, CA, USA).

### Statistical analysis

Sample size was determined by power analysis using a significance level of α = 0.05 with 80% power to detect significant differences. Power analysis and sample size calculations were performed using SAS 9.1 software (SAS Institute Inc., Cary, NC, USA). The experimental design was based on previous publications with similar mechanistic studies (20, 28–30). All results were analyzed by investigators blinded to the treatment. All data are shown as means ± sem. Statistical analyses were performed with Prism 6.0 software (GraphPad, La Jolla, CA, USA). Two-tailed, unpaired Student’s *t* test was used to determine significance of differences between 2 groups. One-way ANOVA followed by the Tukey *post hoc* test were used for comparison of multigroup data. Values of *P* < 0.05 were considered significant.

## RESULTS

### Up-regulated TSPO expression after ICH in humans and mice

**Figure 1.**
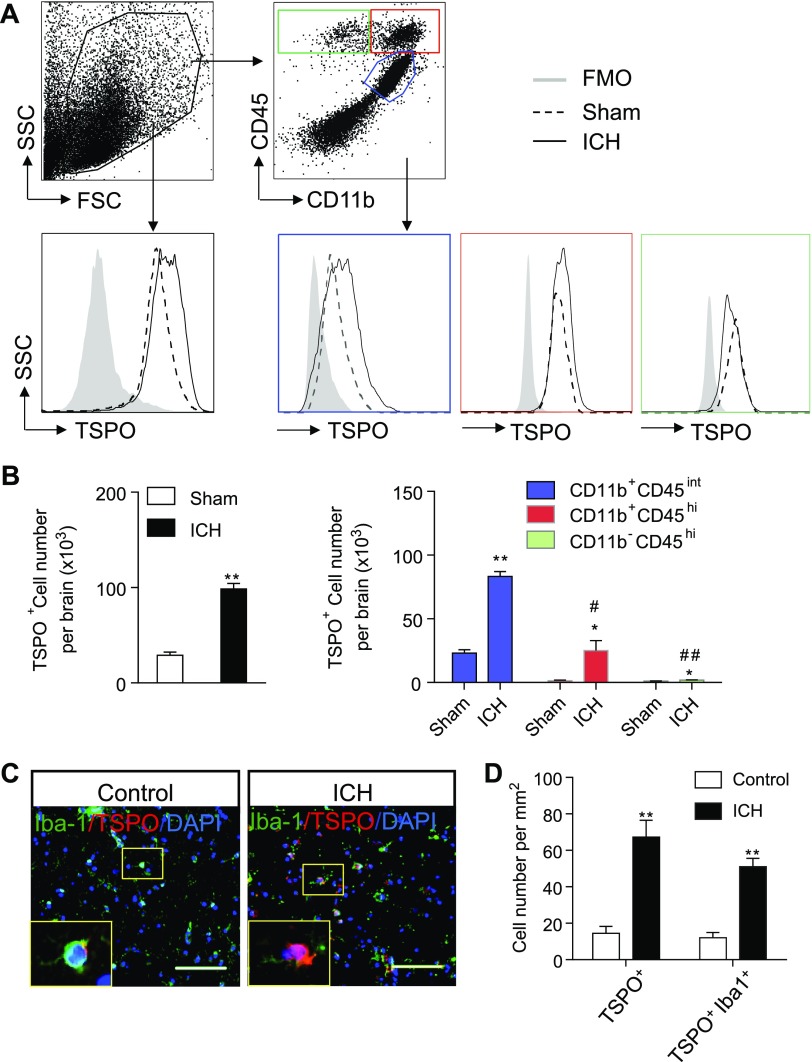
Up-regulation of TSPO in the brains of ICH mice and patients with ICH. *A*) Single-cell suspensions were prepared from brain tissues of mice with ICH induced by collagenase injection at 24 h after surgery. Flow cytometry plots show gating of CD11b^+^CD45^int^, CD11b^+^CD45^hi^, and CD11b^-^CD45^hi^ cell subsets that express TSPO. *B*) Bar graph shows the expression of TSPO in indicated cell subsets (*n =* 6 per group). *P < 0.05, **P < 0.01 *vs.* sham-treatment group of each cell subset. ^#^*P* < 0.05, ^##^*P* < 0.01 *vs.* ICH group of CD11b^+^CD45^int^ cell subsets. *C*, *D*) Immunostaining (*C*) and summarized results (*D*) of TSPO and Iba1 in brain sections from patients with ICH (<24 h after onset) or nonneurologic disease controls. Scale bars, 100 μm (*n =* 20 sections from 5 patients with ICH; *n =* 15 sections from 4 control subjects). Throughout, data are presented as means ± sem. **P < 0.01 *vs.* control group of each cell subset.

In the determination of which immune cells might express TSPO after ICH, we prepared single-cell suspensions of brain tissues from mice injected with collagenase to induce ICH and measured the expression profile of TSPO using flow cytometry. We found a marked increase in cells expressing TSPO after ICH (**Fig. 1*A***, ***B***). Of note, CD11b^+^CD45^int^ microglia were the most prominent immune cell type that expressed TSPO 24 h after ICH. In contrast, the expression of TSPO was less abundant in CD11b^+^CD45^hi^ myeloid cells or barely seen in infiltrating CD11b^−^CD45^hi^ leukocytes. In addition, immunostaining analysis revealed that TSPO expression was significantly up-regulated in Iba-1^+^ cells, including microglia in the brain sections from patients with ICH, as compared to the controls (*i.e.,* tissues from patients with nonneurologic diseases) (Fig. 1*C*, *D*). Together, these data suggest a marked up-regulation of TSPO in brain microglia after ICH onset.

### Etifoxine reduces neurodeficits and brain edema after ICH

**Figure 2.**
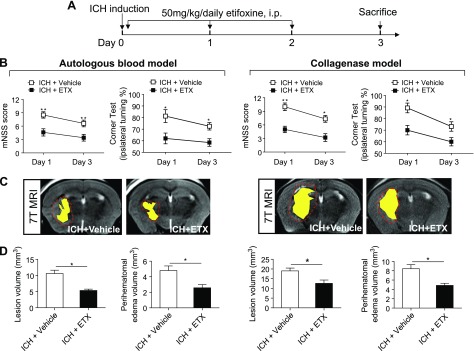
Etifoxine attenuated brain injury in 2 mouse models of ICH. *A*) Flow chart illustrates the regimen of TSPO administration and experimental design. ICH was induced in mice by injection of autologous blood or collagenase, immediately followed by intraperitoneal injection of etifoxine (ETX; 50 mg/kg) or vehicle, which was continued daily until the end of the experiment. *B*) A battery of neurologic tests was performed to evaluate the motor, sensory, and balance functions in the mice given vehicle or ETX at d 1 and after ICH induced by collagenase injection or autologous blood injection. *C*, *D*) Sequential 7-T MRI was used to visualize and measure lesion, hematoma, and edema volumes 3 d after ICH. *C*) T2-weighted image (T2WI) sequences were scanned to assess lesion volume, as outlined with the red line. SWI sequences were assessed for hematoma lesion volumes, visible in yellow regions. Representative 7-T MRI imaging of these lesions and hematomas resulting from ICH were induced by injections of either collagenase or autologous blood. *D*) Quantification of lesion volume and edema volume in mice given vehicle or etifoxine treatment 3 d after ICH induced by injection of either collagenase or autologous blood. Data are means ± sem (*n =* 10 mice per group). *P < 0.05, **P < 0.01 *vs.* control group at indicated time points.

To assess whether the TSPO ligand, etifoxine, affects brain injury after ICH, we examined neurodeficits, lesion volume, and perihematomal edema in ICH mice receiving etifoxine or a vehicle control. Etifoxine (50 mg/kg) or vehicle (1% Tween-80) was injected intraperitoneally for 3 consecutive days starting immediately after the injection of autologous blood or collagenase (**Fig. 2*A***). Neurodeficits were measured using the mNSS and the corner turning test at d 1 and 3 after ICH induction in both models. Lesion volume and hematoma volume were evaluated with T2 and SWI MRI images 3 d after ICH induction. As a result, etifoxine treatment significantly reduced neurodeficits and perihematomal brain edema after the onset of ICH (Fig. 2*B*–*D*). In addition, etifoxine reduced water content in the brain after ICH (Supplemental Fig. 1*A*, *B*). Etifoxine was protective in a dose-dependent manner among the doses examined (25–100 mg/kg). We elected to use the dose of 50 mg/kg in this study, because etifoxine at this dose can provide efficacy similar to that of 100 mg/kg.

### Etifoxine alleviates leukocyte infiltration and microglial production of proinflammatory cytokines after ICH

**Figure 3.**
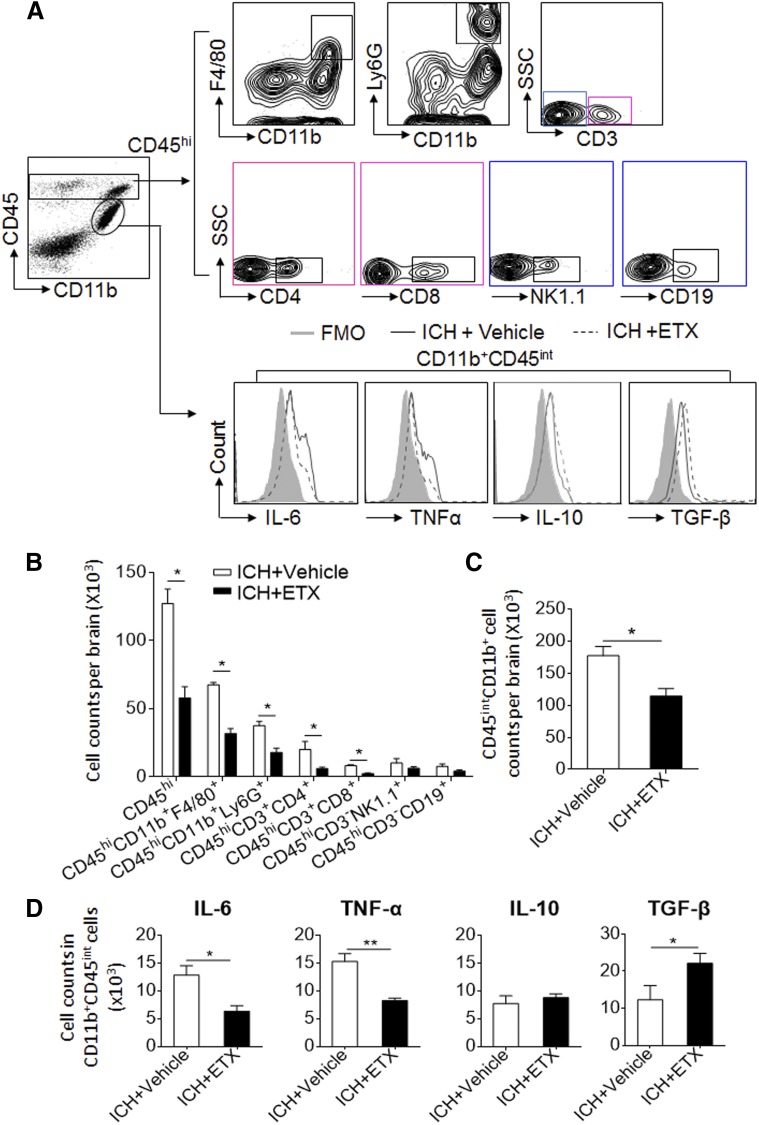
Etifoxine reduced cellular components and microglial proinflammatory cytokine production in the brain after ICH. ICH was induced by collagenase injection and immediately followed by daily intraperitoneal injection of etifoxine (ETX, 50 mg/kg) or vehicle until the end of the experiment. Three days after injection, brain tissues were harvested to isolate single cells for flow cytometry analysis. *A*) The gating strategy for isolating microglia (CD11b^+^CD45^int^), CD4^+^ T cells (CD45^hi^CD3^+^CD4^+^), CD8^+^ T cells (CD45^hi^CD3^+^CD8^+^), NK cells (CD45^hi^CD3^-^NK1.1^+^), neutrophils (CD45^hi^CD11b^+^1A8^+^), and macrophages (CD45^hi^CD11b^+^ F4/80^+^). *B*, *C*) Cell counts of microglia and CNS-invading leukocytes in the brain after ICH. *D*) Cell counts of microglia expressing IL-6, TNFα, IL-10, and TGF-β in brains from ICH mice given vehicle or etifoxine treatment (*n =* 6 per group). Data are means ± sem. *P < 0.05, **P < 0.01.

We then sought to determine the impact of etifoxine on brain inflammation after ICH. For that purpose, we used flow cytometry to measure cellular components in the ICH-afflicted brain including brain-infiltrating leukocytes and microglia (**Fig. 3*A***). We found that etifoxine treatment significantly reduced the counts of brain-infiltrating macrophages (CD45^hi^CD11b^+^F4/80^+^), neutrophils (CD45^hi^CD11b^+^Ly6G^+^), CD4^+^ T cells (CD45^hi^CD3^+^CD4^+^), CD8^+^T cells (CD45^hi^CD3^+^CD8^+^), B cells (CD45^hi^CD3^-^CD19^+^), NK cells (CD45^hi^CD3^-^NK1.1^+^) and microglia (CD11b^+^CD45^int^) (Fig. 3*B*, *C*). In addition, decreased expression of IL-6 and TNF-α and increased expression of TGF-β were seen in microglia after etifoxine treatment (Fig. 3*D*). These results suggest that etifoxine can reduce brain inflammation after ICH.

### Etifoxine attenuates BBB damage and cell death after ICH

**Figure 4.**
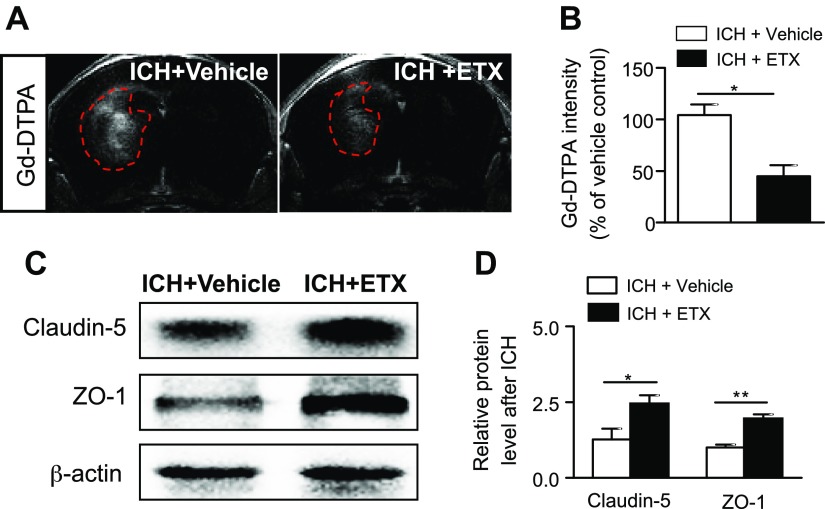
Etifoxine attenuated BBB leakage and loss of tight junction proteins after ICH. ICH was induced by collagenase injection and immediately followed by daily intraperitoneal injection of etifoxine (ETX, 50 mg/kg) or vehicle until the end of the experiment. Three days after injection, sequential 7-T MRI scans were assessed for BBB leakage into the brain, and Western blot analysis was used to detect tight junction protein expression. *A*, *B*) Seven-tesla MRI (*A*) and quantification of data (*B*) show that etifoxine reduced Gd-DTPA extravasation to brain parenchyma. T1-weighted image (T1) sequences were scanned for this evaluation. Red dashed lines: region of Gd-DTPA leakage (*n =* 6 per group). *C*, *D*) Western blot analysis (*C*) and quantification of data (*D*) show that etifoxine preserves tight junction protein (claudin-5 and ZO-1) expression. Data are means ± sem (*n =* 4 per group). *P < 0.05, **P < 0.01.

**Figure 5.**
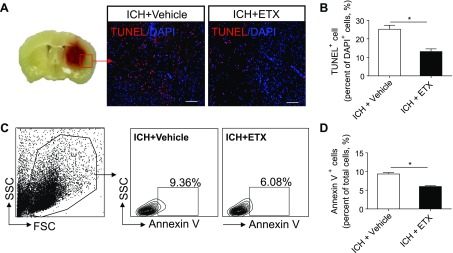
Etifoxine reduced cell death in the brain after ICH. ICH was induced by collagenase injection and immediately followed by daily injections of etifoxine (ETX, 50 mg/kg, i.p.) or vehicle until the end of the experiment. Three days after injection, brain tissues of ICH mice receiving etifoxine or vehicle treatment were harvested for cell death analysis. *A*) Brain tissue sections were stained with TUNEL to measure cell death. Representative images show TUNEL^+^ cells in ICH mice receiving etifoxine or vehicle control. Red rectangle indicates the measured region. Graphs show quantified data. Scale bars, 100 μm. *B*) Summarized results show reduced cell death in ICH mice receiving etifoxine treatment (*n =* 6 per group). *C*, *D*) Flow cytometry plots (*C*) and summarized results (*D*) show annexin V^+^ cells in brain tissues from the indicated groups (*n =* 6 per group). Data are means ± sem. *P < 0.05.

The dysregulated BBB function after ICH contributes to vasogenic edema and perihematomal edema development (4, 31). To determine whether etifoxine impacts BBB integrity after ICH, we measured BBB permeability and expression of tight junction proteins. The presence of brain parenchymal enhancement on contrast-enhanced T1 is generally accepted as an indicator of contrast medium leakage across the disrupted BBB. The quantification of parenchymal enhancement showed a significant decrease in ICH mice receiving etifoxine *vs.* vehicle controls (**Fig. 4*A***, ***B***). Similarly, etifoxine-treated ICH mice had far less Evans Blue extravasation than recipients of the vehicle control (Supplemental Fig. 2*A*, *B*). Western blot measurement showed that etifoxine preserved the expression of the tight junction proteins claudin-5 and ZO-1 after ICH (Fig. 4*C*, *D*). In addition, immunostaining and flow cytometry analyses were performed to determine cell death. Quantitation of TUNEL^+^ cells in the perihematomal area or annexin V^+^ cells in the brain portrayed the reduction of cell apoptosis after etifoxine treatment (**Fig. 5**). Together, these results suggest that etifoxine can preserve BBB integrity after ICH.

### Microglia contribute to the protective effect of etifoxine

**Figure 6.**
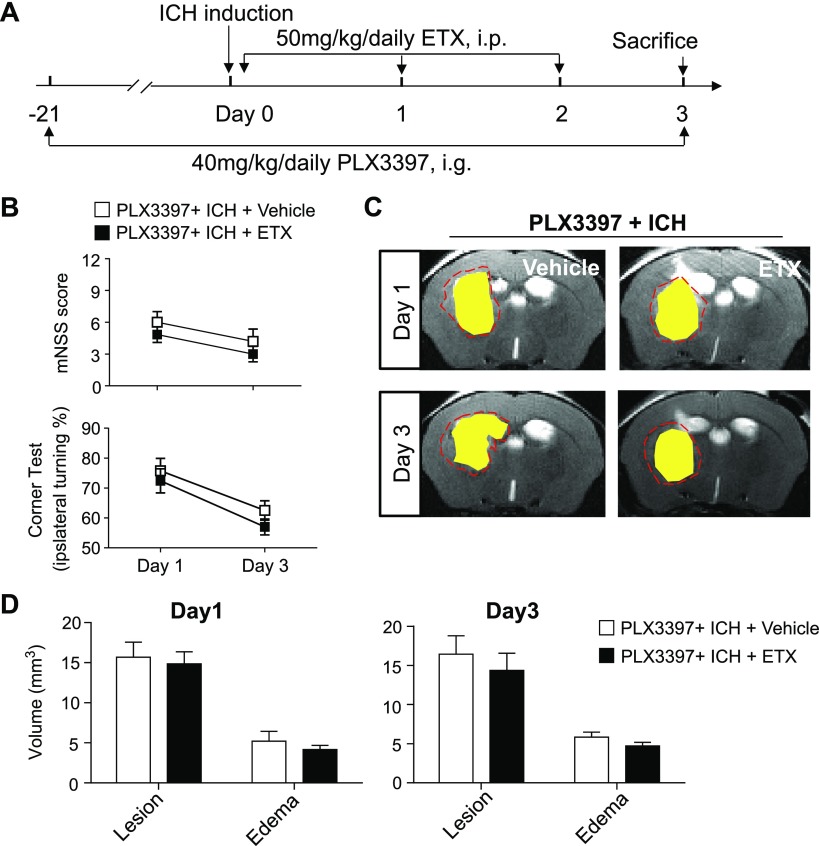
The protective effect of etifoxine was abolished in ICH mice depleted of microglia. Mice were treated with PLX3397 (40 mg/kg) before ICH surgery for 21 d, and the treatment continued until the experiment ended. ICH was induced by collagenase injection. Etifoxine injections started immediately after ICH induction and continued daily until d 2. *A*) Flow chart illustrates the regimen of PLX3397 and etifoxine treatment and ICH induction. *B*) Neurodeficit assessment shows mNSS and corner-turning test score in ICH mice depleted of microglia subjected to etifoxine or vehicle treatment. *C*, *D*) MRI (*C*) and quantification of lesion volume and perihematomal edema volume in indicated groups of mice (*D*) at d 1 and 3 after ICH (*n =* 15 per group). Data are presented as means ± sem.

Our previous study showed that microglia have a detrimental effect on the brain during the injury phase of ICH (20). Because the expression of TSPO occurs primarily in microglia after ICH, we sought to determine whether etifoxine provides protection by targeting microglia. The survival of microglia depends on signaling through colony-stimulating factor 1 receptor (CSF1R) (20, 32). Using the CSF1R inhibitor PLX3397, we effectively depleted microglia preceding ICH induction. PLX3397 treatment continued in this group of mice until the end of the experiments (**Fig. 6*A***). Flow cytometry measurement of microglia (CD11b^+^CD45^int^) showed that depletion efficacy was ∼90% before or after ICH (20). We found that the protective effect of etifoxine was abolished in mice receiving PLX3397 (Fig. 6*B*–*D*). These results demonstrated that the protection conferred by etifoxine treatment against brain injury after ICH requires microglia.

## DISCUSSION

This study provides, for the first time to our knowledge, evidence that etifoxine attenuates hemorrhagic brain injury. Etifoxine significantly reduced neurodeficits and perihematomal brain edema in 2 models of experimental ICH. Etifoxine treatment was sufficient to reduce leukocyte infiltration, decrease microglial production of proinflammatory factors, improve BBB integrity, and alleviate cell death after ICH. In addition, the protection provided by etifoxine was lost in mice subjected to highly efficient depletion of microglia. These results suggest that etifoxine may be a viable drug candidate for ICH treatment.

Microglial activation occurs within minutes after the onset of ICH, and activated microglia were found in the perihematomal area. Microglia possess multiple capabilities that would have an impact on hemorrhagic brain injury, including production of inflammatory or anti-inflammatory cytokines and antigen presentation (33, 34). Indeed, we and others have demonstrated that microglial activation correlates with observations of perihematomal edema, and the inhibition or depletion of microglia activation can provide neuroprotection after ICH (35, 36), suggesting a detrimental role of microglia in ICH. A recent study showed that TSPO expression appeared mostly in microglia, but not in astrocytes or neurons (9). Consistent with this report, we found low expression of TSPO in uninjured brains in contrast with ICH-induced up-regulation of TSPO in CD11b^+^CD45^int^ microglia. Combined with our data showing the expression of TSPO in IBa1^+^ cells in brain sections from patients with ICH, these results suggest that TSPO may be a suitable target for modulating the microglial response after ICH.

To understand the possible mechanisms by which etifoxine might confer protection after ICH, we examined the immune responses in the brain. Evidence has shown that activated microglia and infiltrating leukocytes are key contributors of neuroinflammation after ICH, because of their rapid release of proinflammatory and immunoactive factors, including cytokines, chemokines, prostaglandins, proteases, and ferrous iron (37–39). In the current study, the administration of etifoxine significantly reduced the infiltration of leukocytes and the production by microglia of proinflammatory factors, together with the preservation of BBB integrity and decreased cell death after ICH. Our results are consistent with those in previous reports showing that TSPO ligands provide neuroprotective effects and limit brain inflammation (12, 14–16). In those studies, reduced expression of the proinflammatory cytokines TNF-α, IL-1β, and IL-6 and improved neural function were seen in multiple models of neural injury (12, 14–16).

Because brain inflammation and BBB dysfunction are key contributors to vasogenic perihematoma formation after ICH, we postulate that etifoxine may mitigate the brain’s inflammatory milieu to confer protection in ICH. In support of this notion, we found that the benefit of etifoxine was abolished in mice depleted of microglia by using a CSF1R inhibitor. Together with the finding that the expression of TSPO primarily occurs in microglia after ICH, this result suggests that the protective effect of etifoxine involves its action on microglia to offset the damage from ICH. Possibly, then, the reduced infiltration of leukocytes results from the etifoxine-induced restriction of microglial responses, which have been reported to enhance neuroinflammation by recruiting and activating lymphocytes (40, 41). However, also noteworthy is the finding that CD11b^+^CD45^hi^ leukocytes, which may include monocytes and macrophages, also express TSPO after ICH onset, albeit less abundantly than microglia. In addition, etifoxine may exert an effect on these CD11b^+^CD45^hi^ leukocytes that restricts brain inflammation. Nevertheless, the precise operating mechanisms by which etifoxine restrict brain inflammation after ICH require further investigation.

Reportedly, etifoxine is a potent enhancer of neurosteroid synthesis (8). Etifoxine-induced production of pregnenolone, progesterone, and allopregnanolone can confer neuroprotection (42–45). Progesterone treatment after ICH in rodents has, indeed, reduced brain edema, inflammatory response and cell death after ICH. Together with those results, our findings suggest that protection by the TSPO ligand etifoxine in ICH may involve the local production of neurosteroids that limit brain inflammation. Of interest, etifoxine is also reported to be a positive allosteric modulator of GABAA receptors (46). As previously documented, a significant decrease in the production of GABA and GABAergic system-related genes was observed in rodent models of ICH (47, 48). Because the GABAA receptor is expressed by microglia/lymphocytes and activation of the GABAA receptor can reduce their proinflammatory activities (49–52), further investigation is needed to better understand whether and to what extent the action of etifoxine on GABAA receptors may contribute to its benefit in ICH.

In summary, our data reveal that etifoxine attenuates brain injury and inflammation after ICH, suggesting that it may serve as a promising candidate for investigation in advanced preclinical ICH studies.

## Supplementary Material

Supplemental Data

## Abbreviations

BBB: blood–brain barrier
CSF1R: colony stimulating factor 1 receptor
Gd-DTPA: gadopentetate dimeglumine
ICH: intracerebral hemorrhage
mNSS: modified neurological severity score
PBR: peripheral-type benzodiazepine receptor
SWI: susceptibility-weighted image
TSPO: translocator protein
