# Supplementary material for: A TSPO ligand attenuates brain injury after intracerebral hemorrhage
Source: FASEB J. 2017 Apr 17;31(8):3278–87. doi: 10.1096/fj.201601377RR (PMC5503714; doi:10.1096/fj.201601377RR)
Supplement: Supplemental Data [file supp_31_8_3278__index.html]

A TSPO ligand attenuates brain injury after intracerebral hemorrhage — Supplemental Data 

# A TSPO ligand attenuates brain injury after intracerebral hemorrhage

## Supplemental Data

- Supplemental Data
