# Supplementary material for: A TSPO ligand attenuates brain injury after intracerebral hemorrhage
Source: FASEB J. 2017 Apr 17;31(8):3278–87. doi: 10.1096/fj.201601377RR (PMC5503714; doi:10.1096/fj.201601377RR)
Supplement: Supplemental Data [file supp_fj.201601377RR_Supplemental_Figures.docx]

**
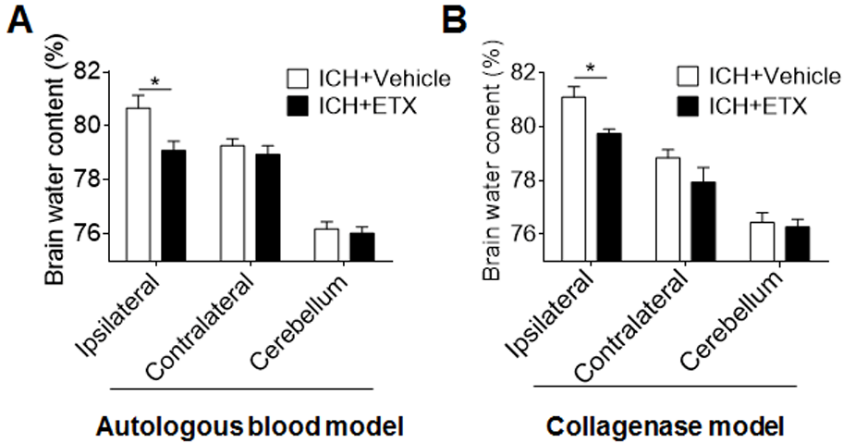
**

**Supplementary Figure 1. Effect of etifoxine on water content in brains of ICH mice.**

ICH was induced by injection of collagenase or autologous blood. **A-B**, Measurement of water content in brains was performed in the ipsilateral hemisphere at day 3 after ICH. *P < 0.05. n = 10 mice per group. Data are presented as mean ± SEM.


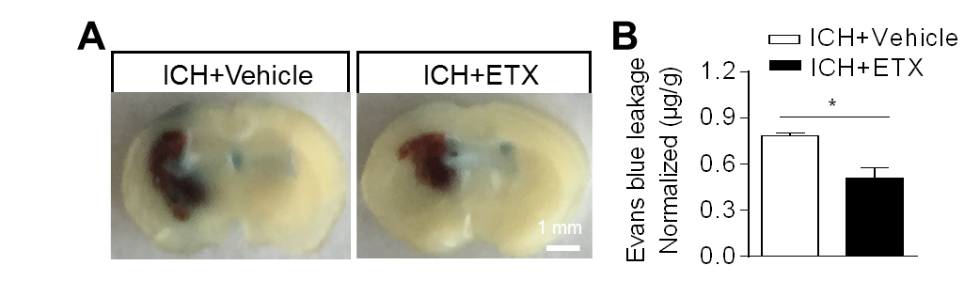


**Supplementary Figure 2. Effect of etifoxine on Evans blue leakage in the brain after ICH.**

ICH was induced by collagenase injection. **A-B**, Evans blue leakage was measured at day 3 after ICH induction in mice receiving the indicated treatment. Scale bar, 1mm. *P < 0.05, n = 6 per group. Data are presented as mean ± SEM.
